# Machine learning–based screening tool for predicting the risk of oropharyngeal dysphagia in patients with ischemic stroke

**DOI:** 10.1007/s00405-026-10515-2

**Published:** 2026-08-05

**Authors:** Suzanne Bettega Almeida, Bianca Marques de Mattos de Araujo, Maria Cristina de Alencar Nunes, Luana Beatriz das Portas Luiz, Rayane Délcia da Silva, Bianca Simone Zeigelboim, Karinna Veríssimo Meira Taveira, Elisa Souza Camargo, Rosane Sampaio Santos, Cristiano Miranda de Araujo

**Affiliations:** 1 Human Communication Health, Center for Artificial Intelligence in Health (NIAS), Tuiuti University of Paraná, Curitiba, Brazil; 2 School of Dentistry, Department of Endodontics, Center for Artificial Intelligence in Health (NIAS), Tuiuti University of Paraná, Street Sydnei Antonio Rangel Santos, 238 - Santo Inacio, Curitiba, Brazil; 3 Center for Artificial Intelligence in Health (NIAS), Federal University of Paraná Hospital Complex - CHC-UFPR - EBSERH, Curitiba, Brazil; 4 School of Dentistry, Tuiuti University of Paraná, Curitiba, Brazil; 5 Speech, Language and Hearing Sciences, Center for Artificial Intelligence in Health (NIAS), Federal University of Rio Grande do Norte, Natal, Rio Grande do Norte Brazil; 6 Pontifícia Universidade Católica do Paraná, Curitiba, Brazil

**Keywords:** Machine learning, Screening, Dysphagia, Ischemic stroke

## Abstract

**Purpose:**

Dysphagia frequently complicates recovery after stroke, necessitating effective predictive tools. This study aimed to develop supervised machine learning models to predict dysphagia risk in ischemic stroke patients.

**Method:**

We retrospectively analyzed data from 103 ischemic stroke patients, aged over 18 years, using fiberoptic endoscopic evaluation of swallowing (FEES) as the diagnostic standard for dysphagia. Clinical variables included age, sex, EAT-10 score, Functional Oral Intake Scale (FOIS^®^), and Glasgow Coma Scale. Eleven ML algorithms were evaluated: Logistic Regression, SVM, Gradient Boosting, Random Forest, Decision Tree, K-Nearest Neighbors, AdaBoost, Multilayer Perceptron, XGBoost, LightGBM, and CatBoost. Data was split into 70% training and 30% testing sets with stratified sampling. Hyperparameter optimization was via grid search and 5-fold stratified cross-validation. Model performance was measured by AUC, accuracy, precision, recall, and F1-score; 95% confidence intervals were estimated by bootstrapping.

**Results:**

Predictive models effectively distinguished dysphagia cases. Gradient Boosting and CatBoost achieved the highest AUC values (AUC = 0.99) on the test set. Logistic Regression performed consistently across test and cross-validation sets, achieving an AUC of 0.95 [95% CI: 0.86–1.00] and precision of 0.91 [95% CI: 0.80–1.00] in the test set, and high values in cross-validation (AUC = 0.93 [95% CI: 0.84–1.00]; precision = 0.92 [95% CI: 0.81–0.99]). Key predictors were FOIS^®^, EAT-10, and age.

**Conclusion:**

Machine Learning models showed promise for dysphagia screening post-stroke, with Gradient Boosting, CatBoost, and Logistic Regression showing strong clinical potential for decision support and early referral.

## Introduction

Stroke is recognized as the leading cause of disability in adults and the second leading cause of death worldwide. According to the World Stroke Organization (WSO), the lifetime risk of stroke has increased by 50% over recent decades [1]. Among the most critical complications of stroke, dysphagia stands out [2], a condition associated with a high risk of pneumonia, particularly in the acute phase of the disease [3].

The prevalence of oropharyngeal dysphagia among post-stroke individuals may reach 60% [4], while its incidence after ischemic stroke ranges from 25.5% to 67% in the first days following the event [5, 6]. Moreover, many patients continue to exhibit dysphagic symptoms months after stroke, even following partial spontaneous recovery of swallowing function [7]. Dysphagia is also associated with serious complications, such as aspiration pneumonia, which significantly increases the risk of in-hospital mortality, with rates reported as high as 70% [8]. Factors such as advanced age, hypertension, diabetes, and brainstem stroke are recognized as independent risk factors for the development of dysphagia [5].

In this context, clinical guidelines recommend that all patients with acute stroke undergo swallowing screening within the first four hours after hospital admission [9]. The healthcare team should use validated protocols and neurological scales to ensure accurate and comprehensive diagnoses [10, 11]. Although advanced techniques, such as fiberoptic endoscopic evaluation of swallowing (FEES), are effective for definitive diagnosis, their use is limited by high costs and the need for trained personnel to perform the procedure and accurately interpret the results [12].

In light of these diagnostic limitations, technological innovation through artificial intelligence (AI), particularly machine learning (ML), has gained increasing prominence in healthcare, enabling detailed analyses of clinical data and the development of effective predictive models [13]. In the specific context of dysphagia, AI has the potential to support the management of this condition, from detection through to rehabilitation [13, 14]. However, the literature remains scarce and heterogeneous, with few studies specifically focused on post-stroke dysphagia. Existing work varies substantially in data types and methodological approaches, including vocalizations converted into spectrograms for remote screening [12], automated videofluoroscopic analysis using deep learning [15], and the use of tabular clinical data to predict the severity of the condition [16], although not always supported by reference diagnostic instruments such as FEES. Therefore, it is essential to conduct new studies employing validated, gold-standard diagnostic methods to ensure greater diagnostic accuracy and enhance the clinical applicability of the developed predictive models.

Unlike previous studies, the present work aimed to develop a predictive model, based on ML algorithms, to estimate the risk of oropharyngeal dysphagia in patients with ischemic stroke using exclusively clinical and functional variables collected prior to instrumental swallowing assessment. Dysphagia status was determined using FEES as the reference diagnostic method. The goal is to provide a targeted, clinically applicable screening tool to support early identification of higher-risk patients in a practical, accessible manner with strong predictive performance.

## Methodology

### Study design

This study was approved by the Research Ethics Committee for Human Subjects (protocol Nº. 2169.064/2010-03). Individual data were obtained from patients admitted to the Stroke Unit of a university hospital in southern Brazil. The original data were collected prospectively between 2024 and 2025. All participants provided written informed consent, and the study was conducted in accordance with the principles of the Declaration of Helsinki.

### Participants

Adults (≥ 18 years) of both sexes were included. Patients required a clinical diagnosis of ischemic stroke (IS) confirmed by a neurologist based on recent brain imaging consistent with the presentation. The National Institutes of Health Stroke Scale (NIHSS) was administered within the first 24 h after symptom onset, and a swallowing assessment was performed within 72 h of IS signs or symptoms. Additionally, participants were required to have a Glasgow Coma Scale (GCS) score of ≥ 11 [17] and no prior speech-language therapy.

Exclusion criteria included prior head and neck surgery, preexisting structural abnormalities of the oropharynx or larynx, brainstem lesions, hemodynamic instability (significant changes in oxygen saturation, respiratory rate, heart rate, body temperature, or blood pressure), and death.

### Data colection

For development of the machine learning predictive model, six variables were collected from each participant. Data collection was conducted by examiners with expertise in dysphagia. The variables used to compose the dataset for the ML model were:

- Glasgow Coma Scale: continuous variable (total score 3–15), based on the assessment of neurological impairment severity [17]. This assessment was conducted by neurologists within 24 h of the ischemic stroke. The GCS evaluates the severity of neurological impairment based on three criteria-eye opening, verbal response, and motor response-assigning scores of 1 to 5, with a total ranging from 3 to 15. Scores are categorized as: normal (15), mild (13–14), moderate (9–12), and severe (3–8).
- Functional Oral Intake Scale (FOIS^®^): categorical variable (levels 1–7), based on the classification of the patient’s functional oral intake ability. The FOIS^®^ assessment was performed by speech-language pathologists before the FEES examination and independently of the endoscopic findings. The evaluators responsible for the FOIS^®^ classification did not have access to the FEES results at the time of assessment. The FOIS^®^ [18] ranges from level 1 (unable to take any food orally) to level 7 (oral intake without restrictions).
- Eating Assessment Tool (EAT10): categorical variable (item score 0–4), based on a 10-item self-administered questionnaire to identify dysphagia risk. The EAT-10 was administered within the first 24 h after stroke, with item scores ranging from 0 (no problem) to 4 (severe problem). A total score ≥ 3 indicates swallowing risk [19]. The EAT-10 has solid psychometric properties and high reliability. Internal consistency, assessed by Cronbach’s alpha, ranges from 0.84 to 0.96 across different versions [20].
- FEES: binary variable (presence or absence of dysphagia), based on fiberoptic endoscopic evaluation of swallowing. This variable served as the label (dependent variable) to be predicted by the model. Dysphagia was defined based on FEES findings indicating altered swallowing safety and/or efficiency during the examination. For the purposes of the machine learning analysis, the FEES outcome was dichotomized into presence or absence of dysphagia, and dysphagia severity levels were not separately modeled. Dysphagia diagnosis via FEES was performed by otorhinolaryngologists, accompanied by an experienced speech-language pathologist, within 72 h of stroke onset. FEES was conducted after the independent collection of the clinical-functional variables, including FOIS^®^, and the evaluators responsible for the FOIS^®^ classification did not have access to the endoscopic findings at the time of assessment. Procedures followed the Fiberoptic Endoscopic Evaluation of Swallowing protocol, using standardized food consistencies commonly adopted in swallowing assessment protocols.
- A blue dye was applied to enhance contrast with the pink mucosa. FEES was performed using a Laryngostrobe 8020 nasofibroscope equipped with a Sony camera, and examinations were recorded for later analysis. During the assessment, patients were instructed to remain seated, and no anesthetic or vasoconstrictor was used. The fibroscope was inserted through a nostril and advanced into the pharynx, allowing visualization of the tongue base, pharyngeal structures, and salivary stasis. Laryngeal sensitivity was assessed by touching the vocal folds and arytenoid cartilages with the scope, observing glottic adduction and the cough reflex. Tested consistencies were thin liquid, nectar, honey, and pudding, administered in 5 mL, 10 mL, and free sips using standard utensils. If swallowing difficulty, laryngeal penetration, or aspiration occurred, consistencies were adjusted, or the examination was halted. The assessment was discontinued in cases of nausea, vomiting, or other signs of clinical instability.
- Sex: categorical variable (male/female), collected via participant self-report.
- Age: continuous variable, expressed in years, based on information provided by the patient at enrollment.

### Sample size

The sample size determination was based on metrics obtained from a pilot study. For the calculation, a moderate effect size (Cohen’s d = 0.5) was adopted, along with a one-tailed test, justified by the prior expectation of the behavior of independent variables associated with the presence of dysphagia, a significance level (α) of 5%, and a statistical power (1 – β) of 80%. The calculations were performed using G*Power software (version 3.1.9.2). Consequently, a minimum sample size of 102 participants was estimated for training the predictive model.

### Development of the model

Predictive models were developed using various supervised machine learning algorithms to predict the presence of dysphagia in patients with ischemic stroke. The selection of nine algorithms aimed to encompass different modeling approaches, balancing predictive capability, clinical interpretability, and adaptability to the available data. The algorithms were classified into different categories based on the learning method adopted:

- Tree-based Models: These make predictions through successive and hierarchical divisions of the data. Within this category, the Decision Tree algorithm was used due to its simple structure and ease of interpretation in the clinical context [21].
- Ensemble Models: These combine multiple classifiers to enhance robustness and predictive performance. The Random Forest was selected for employing multiple trees built from random subsets of the data. Meanwhile, Gradient Boosting uses sequential training, where each new model aims to correct the errors made by the previous ones. XGBoost adopts an iterative strategy for minimizing the loss function to enhance prediction accuracy, while AdaBoost dynamically adjusts the weights of samples, placing greater emphasis on instances that are misclassified. Also included were LightGBM, which employs leaf-wise tree growth and histogram-based techniques to increase computational efficiency, and CatBoost, which is notable for its native handling of categorical variables and the incorporation of internal regularization mechanisms that reduce the risk of overfitting [22–25].
- Linear Models: These assume a linear relationship between the predictor variables and the clinical outcome [26]. Logistic Regression was chosen for being widely used in clinical practice, as well as having a simple structure and direct interpretation.
- Instance-based Models: These classify new observations based on similarity to previously labeled examples. The K-Nearest Neighbors (KNN) algorithm, which is non-parametric in nature, considers the k nearest neighbors of a new instance to determine its class, with the option to weight based on distance.
- Artificial Neural Network Models: These simulate nonlinear relationships between variables through multiple layers of processing [27]. In this study, the Multi-layer Perceptron (MLP) was used, a type of feedforward network that incorporates hidden layers and nonlinear activation functions to capture complex patterns [28].
- Margin-based Models: These build hyperplanes that maximize the separation between classes [29]. The Support Vector Machine (SVM) was utilized for its high generalization capability and effectiveness in scenarios with a large number of predictor variables [30].

### Model training, hyperparameter optimization, cross-validation, testing, and overfitting control

The data were randomly divided into two subsets: 70% for model training, allowing the algorithms to identify relevant patterns associated with dysphagia, and the remaining 30% for the testing set, used to assess the predictive capacity of the models on data not utilized during training.

To ensure the best possible performance of each model, hyperparameter optimization was conducted using the grid search technique, which systematically tests different combinations of parameters until the most effective configuration is identified [31]. This process was integrated into a stratified cross-validation strategy with five subdivisions (folds), in which the training data is divided into five parts. The model is then repeatedly trained and validated, each time with a different combination of these parts, allowing for a more consistent evaluation of its performance and helping to reduce the risk of overfitting [32].

To make the analyses more reliable and assess the variability of the performance metrics, the bootstrap method was used with 1,000 resampling. This technique allowed for the calculation of 95% confidence intervals (CI 95%) for both the results from the test set and those obtained during the cross-validation rounds.

### Model evaluation metrics and feature importance

The performance of the models was evaluated using the Area Under the Receiver Operating Characteristic Curve (AUC). The AUC was employed to analyze the model’s discrimination ability, with higher values indicating better performance in distinguishing between patients with and without dysphagia. For this calculation, the roc_auc_score function from the scikit-learn library was used, determining the area under the curve (AUC) representing the overall accuracy of the diagnostic test—higher values suggest superior performance, with an AUC of 1.0 corresponding to a perfect test and an AUC of 0.5 indicating performance equivalent to chance [33]. In addition, other performance metrics of the models were also calculated.

- Accuracy: The proportion of correct predictions relative to the total number of cases evaluated. It indicates the overall efficiency of the model.
- Precision: The proportion of cases with dysphagia correctly classified among all those classified as positive. It assesses the reliability of positive predictions.
- Weighted Recall: The proportion of dysphagia cases correctly identified among all those who actually exhibited the outcome. It measures the model’s ability to recognize patients at risk of dysphagia.
- F1 Score: The harmonic mean between precision and recall. It summarizes the model’s performance by considering both correct identifications and errors in positive predictions.

All results were presented with 95% confidence intervals (CI). The analysis of variable (feature) importance was conducted only for the models that natively provide direct measures of importance. For the tree-based algorithms—Decision Tree, Random Forest, Gradient Boosting, AdaBoost, XGBoost, LightGBM, and CatBoost—the feature_importances_ attribute was used, which estimates the relative contribution of each predictor based on the reduction of impurity during the construction of the trees.

In the case of Logistic Regression, the absolute values of the estimated coefficients (coef_) were used as a measure of the relative influence of each variable on predicting the outcome. Models such as K-Nearest Neighbors (KNN), Support Vector Machine (SVM) with RBF kernel, and Multi-layer Perceptron (MLP) were not included in this step, as they do not natively provide direct measures of variable importance. The variables considered most relevant were those that demonstrated a significant impact across most of the analyzed models, taking into account both the magnitude and the frequency with which they appeared as important.

### Comparison of model performance

The comparison among the predictive models regarding their discriminative ability was conducted through paired analyses based on AUC values, accompanied by their respective 95% confidence intervals obtained via bootstrap resampling with 1,000 iterations. The difference between the models was considered statistically significant when the confidence interval of the difference between the AUCs did not include the value zero.

## Results

### Sample characterization

Of the 144 patients who met the inclusion criteria, 41 (28.5%) were excluded. Among those excluded, five (12.2%) died, and 36 (87.8%) exceeded the 72-hour limit for undergoing the FEES.

The final sample used for the development of the machine learning model consisted of 103 patients diagnosed with ischemic stroke, confirmed by computed tomography. Of these, 39 (37.9%) were female, and 64 (62.1%) were male. The average age was 65.6 ± 13.0 years for women (range: 37–96 years) and 64.4 ± 15.5 years for men (range: 30–90 years). The characterization of the sample can be seen in Table 1.

**Table 1:** Sample Characterizatio

**Variável**	**Categoria**	**n**	**%**
Glasgow Coma Scale	15 (cognitive normality)	57	55,3%
	14 (mild impairment)	20	19,4%
	13 (mild impairment)	15	14,6%
	12 (moderate impairment)	4	3,9%
	11 (moderate impairment))	7	6,8%
EAT-10	No risk for dysphagia	57	55,3%
	Imminent risk for dysphagia	46	44,7%
FOIS^®^	Level 1 (nothing by mouth)	25	24,3%
	Level 2 (alternative route + minimal oral intake)	2	1,9%
	Level 4 (oral intake of a single consistency)	6	5,8%
	Level 5 (multiple consistencies with modifications)	10	9,7%
	Level 6 (multiple consistencies with restrictions)	1	1,0%
	Level 7 (total oral intake without restrictions)	59	57,3%
FEES (Dysphagia)	Absent	57	55,3%
	Present	46	44,7%

### Predictive importance of variables

**Fig. 1:**
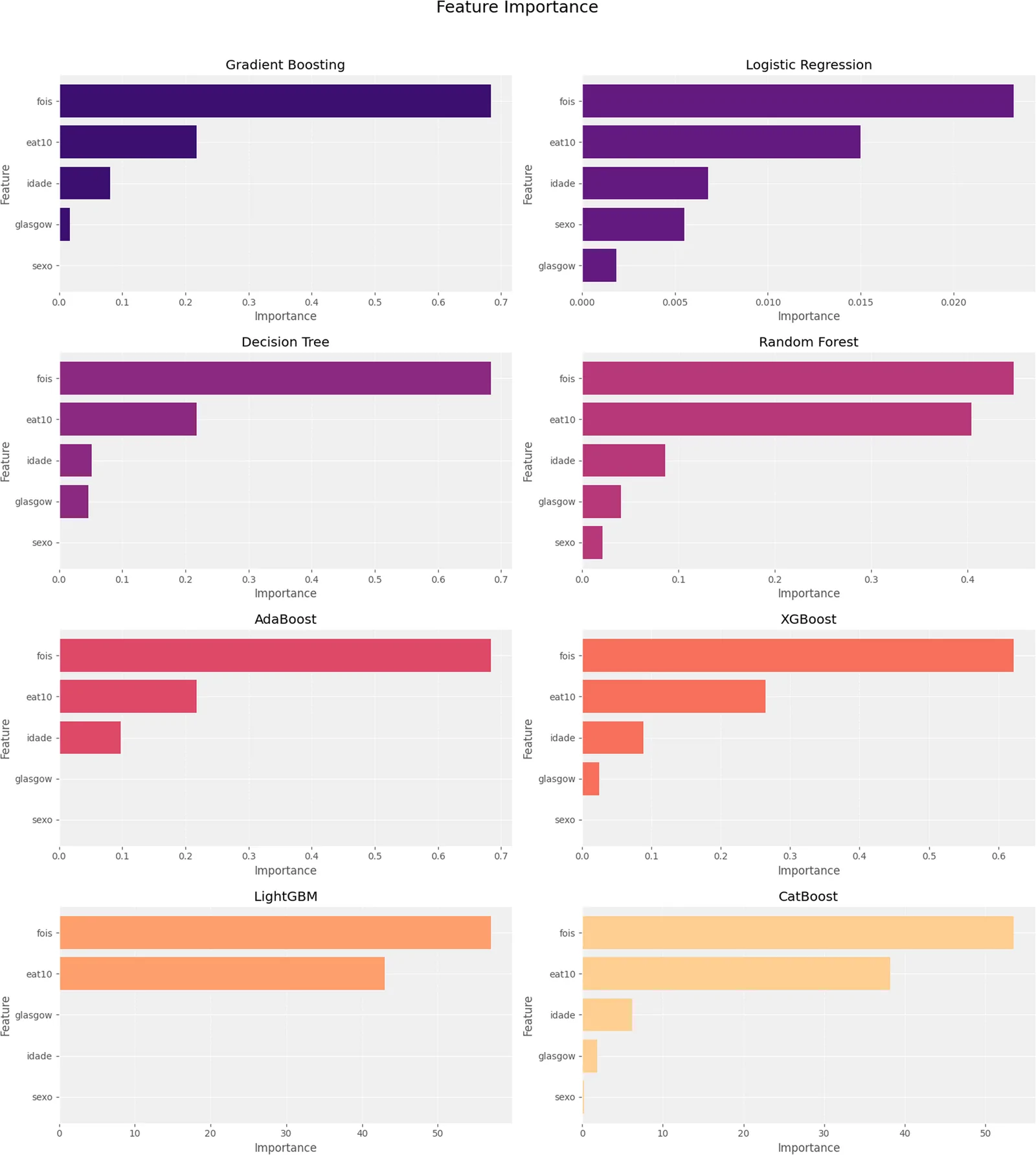
Graph of Predictive Importance of Variables Used in the Model

Six clinical variables were considered in the construction of the dataset used for the development of the machine learning models. Among them, five were used as predictor variables: sex, age, score on the Glasgow Coma Scale, EAT-10 score, and functional feeding level according to the FOIS^®^. The diagnosis of dysphagia, determined through the FEES, was adopted as the dependent variable. Among the predictor variables, the functional feeding level (FOIS^®^) demonstrated the highest predictive power across all evaluated models, followed by the EAT-10 scores and age (Fig. 1).

### Model performance

**Fig. 2:**
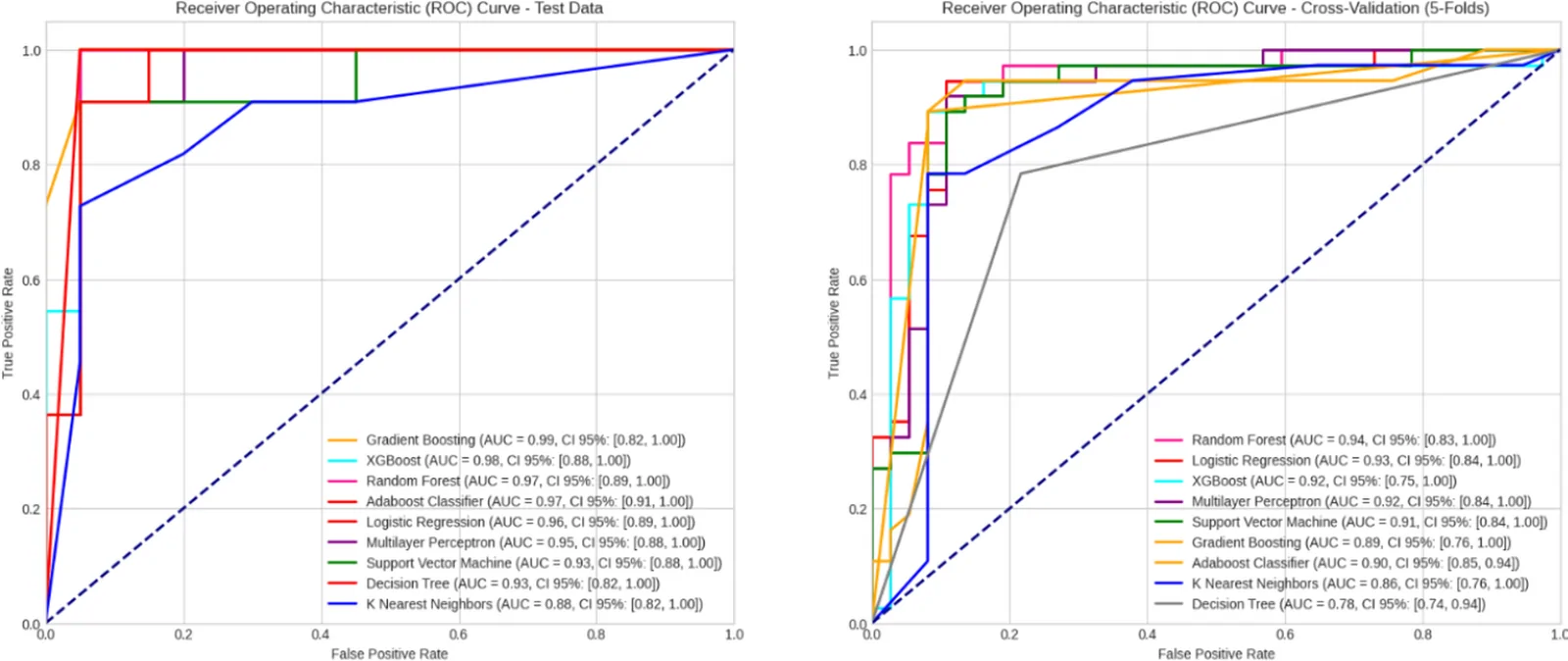
ROC Curve of Predictive Models for Dysphagia Risk in Patients with Ischemic Stroke

The models that demonstrated superior performance in terms of AUC on the test data were Gradient Boosting and CatBoost, both with an AUC of 0.99, and precision of 0.94 [CI 95% = 0.84–1.00] and 0.97 [CI 95% = 0.92–1.00], respectively. The XGBoost and Random Forest models also exhibited high discriminative power, with AUCs of 0.98 and 0.97, and precision values of 0.97 [CI 95% = 0.92–1.00]. In cross-validation, the Logistic Regression and SVM models achieved the highest AUC values, with precision of 0.92 [CI 95% = 0.81–0.99] and 0.90 [CI 95% = 0.80–0.99], respectively. The Gradient Boosting and LightGBM models also demonstrated consistent performance, with AUCs of 0.89 and 0.90 and precision values of 0.88 [CI 95% = 0.80–0.94] and 0.89 [CI 95% = 0.80–0.94], respectively (Fig. 2).

Although the Gradient Boosting and CatBoost models showed the highest AUC values on the test data (both with AUC = 0.99), the most consistent performance between the test data and cross-validation was observed in the Logistic Regression model. This model achieved an AUC of 0.95 [CI 95% = 0.86–1.00] and precision of 0.91 [CI 95% = 0.80–1.00] on the test, maintaining high performance in cross-validation, with an AUC of 0.94 [CI 95% = 0.84–1.00] and precision of 0.92 [CI 95% = 0.81–0.99]. The SVM model exhibited similar behavior, with an AUC of 0.93 [CI 95% = 0.88–1.00] on the test and 0.94 [CI 95% = 0.82–1.00] in cross-validation, along with precision values of 0.91 [CI 95% = 0.80–1.00] and 0.90 [CI 95% = 0.80–0.99], respectively. The performance of all models and the corresponding hyperparameters can be viewed in Table 2.

**Table 2:** Metrics for Cross-Validation and Testing for Each Model, with Corresponding 95% Confidence Intervals

Model	Optimal Hyperparameters	Test Data Results [CI 95%]	Cross-Validation Results [CI 95%]
Gradient Boosting	‘learning_rate’: 0.01, ‘max_depth’: 3, ‘min_samples_leaf’: 1, ‘min_samples_split’: 2, ‘n_estimators’: 50	Accuracy: 0.94 [0.84–1.00] Precision: 0.94 [0.84–1.00] Recall: 0.94 [0.84–1.00] F1-Score: 0.94 [0.84–1.00] AUC: 0.99 [0.82–1.00]	Accuracy: 0.88 [0.79–0.93] Precision: 0.88 [0.80–0.94] Recall: 0.88 [0.79–0.93] F1-Score: 0.88 [0.79–0.93] AUC: 0.89 [0.79–0.98]
Logistic Regression	‘C’: 0.001, ‘l1_ratio’: 0.2, ‘max_iter’: 50, ‘penalty’: ‘l2’, ‘solver’: ‘liblinear’	Accuracy: 0.90 [0.77–1.00] Precision: 0.91 [0.80–1.00] Recall: 0.90 [0.77–1.00] F1-Score: 0.90 [0.78–1.00] AUC: 0.95 [0.86–1.00]	Accuracy: 0.90 [0.79–0.99] Precision: 0.92 [0.81–0.99] Recall: 0.90 [0.79–0.99] F1-Score: 0.90 [0.79–0.99] AUC: 0.94 [0.84–1.00]
SVM	‘C’: 0.9, ‘gamma’: ‘auto’, ‘kernel’: ‘linear’	Accuracy: 0.90 [0.77–1.00] Precision: 0.91 [0.80–1.00] Recall: 0.90 [0.77–1.00] F1-Score: 0.90 [0.78–1.00] AUC: 0.93 [0.88–1.00]	Accuracy: 0.89 [0.79–0.99] Precision: 0.90 [0.80–0.99] Recall: 0.89 [0.79–0.99] F1-Score: 0.89 [0.79–0.99] AUC: 0.94 [0.82–1.00]
KNN	‘leaf_size’: 1, ‘n_neighbors’: 7, ‘p’: 0.001	Accuracy: 0.93 [0.84–1.00] Precision: 0.94 [0.84–1.00] Recall: 0.93 [0.84–1.00] F1-Score: 0.93 [0.84–1.00] AUC: 0.93 [0.80–1.00]	Accuracy: 0.85 [0.72–0.99] Precision: 0.87 [0.75–0.99] Recall: 0.85 [0.72–0.99] F1-Score: 0.85 [0.72–0.99] AUC: 0.94 [0.87–1.00]
Decision Tree	‘criterion’: ‘gini’, ‘max_depth’: None, ‘splitter’: ‘best’	Accuracy: 0.81 [0.68–0.94] Precision: 0.82 [0.68–0.94] Recall: 0.81 [0.68–0.94] F1-Score: 0.81 [0.67–0.94] AUC: 0.93 [0.82–1.00]	Accuracy: 0.81 [0.73–0.92] Precision: 0.82 [0.74–0.93] Recall: 0.81 [0.73–0.92] F1-Score: 0.81 [0.73–0.92] AUC: 0.80 [0.72–0.92]
Random Forest	‘criterion’: ‘entropy’, ‘max_depth’: None, ‘max_features’: ‘sqrt’, ‘min_samples_leaf’: 4, ‘min_samples_split’: 2	Accuracy: 0.97 [0.90–1.00] Precision: 0.97 [0.92–1.00] Recall: 0.97 [0.90–1.00] F1-Score: 0.97 [0.90–1.00] AUC: 0.97 [0.89–1.00]	Accuracy: 0.84 [0.74–0.91] Precision: 0.85 [0.75–0.92] Recall: 0.84 [0.74–0.91] F1-Score: 0.84 [0.73–0.91] AUC: 0.95 [0.85–1.00]
AdaBoost	‘learning_rate’: 0.01, ‘n_estimators’: 5	Accuracy: 0.81 [0.65–0.94] Precision: 0.85 [0.72–0.95] Recall: 0.81 [0.65–0.94] F1-Score: 0.81 [0.66–0.94] AUC: 0.83 [0.82–1.00]	Accuracy: 0.84 [0.74–0.93] Precision: 0.84 [0.74–0.94] Recall: 0.84 [0.74–0.93] F1-Score: 0.83 [0.74–0.93] AUC: 0.83 [0.73–0.93]
XGBoost	‘learning_rate’: 0.1, ‘max_depth’: 3, ‘n_estimators’: 200	Accuracy: 0.97 [0.90–1.00] Precision: 0.97 [0.92–1.00] Recall: 0.97 [0.90–1.00] F1-Score: 0.97 [0.90–1.00] AUC: 0.98 [0.91–1.00]	Accuracy: 0.82 [0.80–0.86] Precision: 0.83 [0.79–0.86] Recall: 0.82 [0.79–0.86] F1-Score: 0.82 [0.79–0.86] AUC: 0.88 [0.81–0.96]
LightGBM	‘learning_rate’: 0.01, ‘max_depth’: 3, ‘n_estimators’: 100	Accuracy: 0.90 [0.78–1.00] Precision: 0.91 [0.81–1.00] Recall: 0.90 [0.78–1.00] F1-Score: 0.90 [0.78–1.00] AUC: 0.98 [0.90–1.00]	Accuracy: 0.87 [0.79–0.93] Precision: 0.89 [0.80–0.94] Recall: 0.87 [0.79–0.93] F1-Score: 0.87 [0.79–0.93] AUC: 0.90 [0.82–1.00]
CatBoost	‘depth’: 6, ‘iterations’: 100, ‘learning_rate’: 0.1	Accuracy: 0.97 [0.90–1.00] Precision: 0.97 [0.92–1.00] Recall: 0.97 [0.90–1.00] F1-Score: 0.97 [0.90–1.00] AUC: 0.99 [0.93–1.00]	Accuracy: 0.86 [0.79–0.93] Precision: 0.87 [0.80–0.94] Recall: 0.86 [0.79–0.93] F1-Score: 0.86 [0.79–0.93] AUC: 0.91 [0.86–0.98]
MLP	‘activation’: ‘relu’,’alpha’: 1.0, ‘hidden_layer_sizes’: (10,), ‘max_iter’: 50, ‘solver’: ‘adam’	Accuracy: 0.90 [0.77–1.00] Precision: 0.90 [0.80–1.00] Recall: 0.90 [0.77–1.00] F1-Score: 0.90 [0.78–1.00] AUC: 0.94 [0.87–1.00]	Accuracy: 0.86 [0.79–0.99] Precision: 0.87 [0.80–0.99] Recall: 0.87 [0.79–0.99] F1-Score: 0.86 [0.79–0.99] AUC: 0.89 [0.78–1.00]

**Fig. 3:**
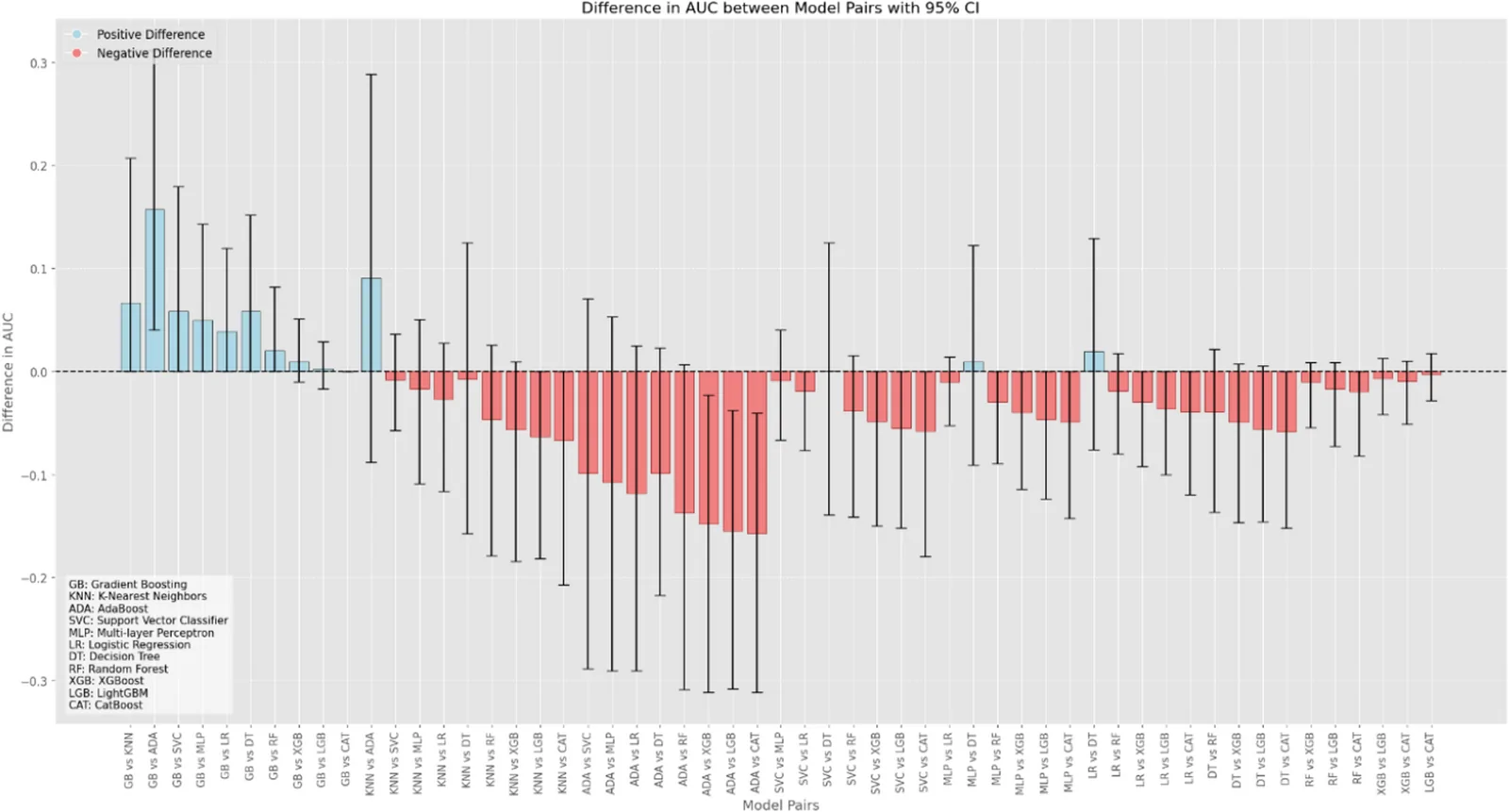
Pairwise Comparison of AUC Differences Between Models, with Corresponding 95% Confidence Intervals (CI 95%)

The AdaBoost model exhibited the poorest performance, with a statistically significant difference compared to four of the tested models (Fig. 3).

## Discussion

Dysphagia is a condition commonly observed in patients affected by stroke, emphasizing the importance of early identification to prevent potential associated clinical complications [4]. The selection of a reliable tool to screen for changes in swallowing presents a challenge, given the inter- and intra-individual variability of the swallowing process, especially in stroke patients, who often develop adaptive and compensatory mechanisms over time [8].

In this context, the present study aimed to develop a predictive model based on machine learning algorithms for the screening of dysphagia in patients with ischemic stroke. The models demonstrated good performance, with high accuracy rates. The variables FOIS^®^, EAT-10, and age emerged as the most relevant predictors. However, the additional value of the machine learning models lies not only in identifying clinically relevant variables, but also in their ability to integrate multiple clinical and functional predictors simultaneously, model complex relationships between variables, and generate individualized risk predictions with high discriminative performance. This approach may contribute to greater standardization and scalability of dysphagia screening, particularly in healthcare settings with limited access to specialized swallowing assessment. Although FOIS^®^ emerged as the most important predictor across the models, this variable was collected before the FEES examination and independently of the endoscopic findings. Therefore, FOIS^®^ was incorporated as a clinical-functional screening variable rather than as part of the diagnostic definition of dysphagia. While FOIS^®^ and FEES evaluate related aspects of swallowing function, the former reflects functional oral intake in routine clinical assessment, whereas FEES was adopted as the instrumental reference standard for outcome definition. This approach aimed to simulate a clinically applicable screening scenario using information available prior to instrumental swallowing assessment.

The integration of functional assessment conducted by speech-language pathologists and instrumental evaluation through techniques such as videofluoroscopic Swallowing Study and Fiberoptic Endoscopic Evaluation of Swallowing can provide a more comprehensive diagnosis [14, 34]. However, several limiting factors must be considered. At an individual level, some patients may have difficulties following instructions during the exams due to postural changes, cognitive impairment, or overall clinical condition. Furthermore, it is necessary to weigh the discomforts associated with the use of the fibroscopic probe, the administration of contrast in the FEES, and radiation exposure during the VFSS [8]. From an organizational perspective, the shortage of qualified professionals to perform and interpret these exams is notable, as well as procedural restrictions related to the availability of equipment, particularly in settings caring for patients with acute stroke. Finally, the financial impact on the healthcare system also warrants attention, considering the costs involved in acquiring equipment and training teams, as well as the need to evaluate the rational allocation of these resources in light of other priority demands [8]. Thus, tools based on artificial intelligence present themselves as promising alternatives to assist in the initial clinical screening of patients suspected of dysphagia. The binary classification strategy adopted in the present study reflects the intended clinical application of the model as a screening tool to support early identification and referral of patients at risk for dysphagia rather than as a definitive diagnostic system for dysphagia severity stratification. Predictive models developed with machine learning algorithms can serve as clinical decision support, facilitating faster identification of at-risk cases, optimizing resource allocation, and expanding access to care, especially in contexts with limited infrastructure or unavailability of instrumental exams.

In clinical practice, healthcare professionals often use bedside swallowing screening instruments to early identify patients at risk of dysphagia [8]. These screening procedures aim to detect health conditions that are unrecognized in asymptomatic populations, using simple and quick-to-apply methods such as questionnaires, clinical observations, and physical examinations [35]. Among these tools, validated self-report questionnaires have gained prominence as they allow direct access to patients’ subjective perception of their symptoms [36]. However, it is important to recognize the limitations of these instruments, particularly regarding the influence of subjectivity from both the patient and the evaluator. This concern is supported by studies discussing widely used instruments, such as the Gugging Swallowing Screen (GUSS), which is described as easy to apply but has significant drawbacks: invasiveness, risk of aspiration, the necessity for a trained administrator, and a high rate of false positives, which can lead to unnecessary referrals [37, 38]. Although the literature presents a wide range of instruments for dysphagia screening and assessment, the use of non-validated methods may lead to overestimation of the prevalence of the condition and compromise diagnostic accuracy [9, 39]. In this scenario, a key advantage of artificial intelligence is its ability to systematically and objectively integrate multiple clinical, functional, and self-reported variables. This approach helps reduce the influence of subjective judgments and enhance the screening process, facilitating more precise and evidence-based clinical decisions.

The use of technology-based devices and systems has emerged as a contemporary strategy to support the analysis and monitoring of swallowing-related functions. The application of machine learning models as a screening tool can generate clinically relevant information through the integrated analysis of routinely collected clinical and functional variables that are often interpreted independently in clinical practice. Additionally, these approaches may facilitate, expedite, and potentially reduce the costs associated with dysphagia diagnosis [36]. Regarding clinical assessment protocols, machine learning-based screening tools may offer another important advantage by reducing unnecessary patient exposure to potential aspiration risks during clinical and/or instrumental swallowing examinations [27]. Feng et al. (2024) proposed machine learning models to predict severe dysphagia in patients with ischemic stroke based on the application of the Water Swallowing Test (WST) within the first 72 h after the event. Nineteen clinical features were considered, and six distinct models were evaluated [13]. However, to the best of our knowledge, the present study is the first to employ machine learning algorithms to predict dysphagia in patients with ischemic stroke using exclusively clinical and functional variables labeled according to a reference diagnostic method recognized as a gold standard (FEES). This approach may contribute to greater reliability of the proposed screening tool and enhance its potential applicability in the clinical context of post-stroke dysphagia.

Overfitting refers to a situation in which a machine learning model becomes excessively adapted to the characteristics of the training dataset, including random variations that are not truly representative of the underlying clinical pattern. As a consequence, the model may exhibit excellent performance during development while showing reduced predictive accuracy and limited generalizability when applied to new data [40]. The literature indicates that this issue is more common in highly flexible or complex algorithms, particularly when the sample size is limited relative to model complexity [40]. In clinical prediction studies, small datasets may also contribute to instability in estimated risks and prediction variability, potentially resulting in optimistic performance estimates and reduced external validity [41, 42]. Therefore, strategies such as cross-validation, bootstrap resampling, penalization methods, and external validation are recommended to improve model robustness and reduce the risk of overly optimistic predictive performance [41]. In the present study, stratified 5-fold cross-validation, grid search hyperparameter optimization, and bootstrap resampling with 1,000 iterations were employed to improve robustness and mitigate overfitting. Although some ensemble-based models achieved extremely high AUC values in the test set, lower performance during cross-validation suggested possible sensitivity to sample-specific patterns. In contrast, Logistic Regression and SVM demonstrated more consistent performance across validation stages, indicating greater stability and robustness despite the limited sample size. Nevertheless, external validation in independent and multicenter populations remains necessary before clinical implementation.

Several limitations of this study should be noted. The predictive model was developed and tested using data from a single institution, which may limit generalizability and contribute to optimistic performance estimates when applied to external populations. Although internal validation strategies, including stratified cross-validation and bootstrap resampling, were employed to mitigate the risk of overfitting and improve model robustness, external validation in independent and multicenter cohorts remains essential before routine clinical implementation. Differences in patient characteristics, institutional protocols, evaluator experience, and healthcare infrastructure may influence model performance across distinct clinical settings. Despite these limitations, the proposal to use a screening tool based on machine learning represents a significant advancement, enabling healthcare professionals, even in the absence of a speech-language pathologist, to early identify patients at high risk for dysphagia. This approach promotes the adoption of more appropriate clinical practices, such as timely referrals for instrumental exams when indicated. Furthermore, it highlights the agility and efficiency of the system in screening for dysphagia in patients with ischemic stroke using initial clinical data, with the added advantage of analyzing complex patterns in large volumes of information, surpassing the inherent limitations of individual human assessment [36, 38, 43].

Future studies should consider the external validation of the model in independent and multicenter populations with different clinical and operational profiles, as well as the assessment of the tool’s applicability in real-time within healthcare services.

## Conclusion

The constructed models demonstrated good performance in predicting dysphagia in patients with ischemic stroke, especially highlighting the Gradient Boosting, CatBoost, and Logistic Regression algorithms. In all models, the FOIS^®^ variable stood out as the most relevant predictor, followed by EAT-10 and age. The application of automated methods based on AI can enable efficient analyses from data that are often underutilized in clinical practice, optimizing dysphagia screening in a quick, accessible manner, and with reduced risk to the patient.

## Data Availability

The data supporting the conclusions of this study are available and can be requested from the corresponding author.
